# Factors Influencing Pre-Cardiopulmonary Arrest Signs among Post-General Surgery Patients in Critical Care Service System

**DOI:** 10.3390/ijerph20010876

**Published:** 2023-01-03

**Authors:** Chunthana Chinawong, Ketsarin Utriyaprasit, Siriorn Sindhu, Chukiat Viwatwongkasem, Sirilak Suksompong

**Affiliations:** 1 Faculty of Nursing, Mahidol University, Bangkok 10700, Thailand; 2 Faculty of Public Health, Mahidol University, Bangkok 10400, Thailand; 3 Faculty of Medicine Siriraj Hospital, Mahidol University, Bangkok 10700, Thailand

**Keywords:** cardiac arrest, pre-cardiopulmonary arrest, intensive care unit, postoperative

## Abstract

Health service system factors can lead to pre-cardiopulmonary arrest signs (pre-CA), which refer to a critical condition in the body leading to a circulatory and respiratory system disruption. The purpose of this study was to assess the incidence rate of an event leading to pre-cardiopulmonary arrest signs within the first 24 h, and also to analyze the factors influencing the health service system in critical post-general surgery patients in the intensive care unit. These results of the study found the incidence rate of pre-CA was 49.05 per 1000 person-hours, especially 1 h after admission to the ICU. Hemodynamic instability, respiratory instability, and neurological alteration were the most common pre-CA symptoms. The patient factors associated with high pre-CA arrest sign scores were the age from 18–40 years, with an operation status as emergency surgery, elective surgery compared with urgent surgery, and the interaction of operation status and age in critical post-general surgery patients. The organization factors found advanced hospital level and nurse allocation were associated with pre-CA. To improve quality of care for critical post-general surgery patients, critical care service delivery should be delegated to nurses with nurse allocation and critical care nursing training. Guidelines must be established for critically ill post-general surgery patient care.

## 1. Introduction

Introduction: In-hospital cardiac arrest had a trend toward preventability because of increased awareness and close monitoring during admission, according to the *American Heart Association Guideline for Cardiopulmonary Resuscitation (CPR) and Cardiovascular Care 2019* [1]. The causes were the disease’s pathology or injuries to the body that affected the body’s circulation and vital organs. The most common primary causes of cardiac arrest (50–60%) were cardiac disease; secondly, it was respiratory insufficiency (15–20%) [2]. Patients admitted to the general surgery intensive care unit (ICU) for undergoing major surgery procedures, such as bowel or gastrointestinal surgery, require general anesthesia and a long period to complete, which may be life-threatening. Most studies report an incidence of one to six events per 1000 hospital admissions [2,3,4,5,6]. The overall incidence of cardiopulmonary resuscitation (CPR) was 1 in 203 surgical cases; however, it varied by specialty (1 in 33 for cardiac surgery vs. 1 in 258 for general surgery). The mortality rate also varied by specialty (45.0–74.5%) [7]. A THAI-ICU study reported the incidence of in-hospital cardiac arrest in the surgical intensive care unit (SICU) as 490 patients per 10,000 surgeries [8].

This is due to the pathology of the disease or injury to the body affecting the circulatory system and vital organs in the body, and its adaptation to the equilibrium, symptoms, and distinctive signs that normally depend on the severity of the disease [9]. Common diseases cause problems in various body systems, such as the rapidly changing circulatory system, respiratory system, nervous system, and the kidney. In addition, the disease severity occasionally affects the organ functions, and then spreads over multiple systems in the body [10,11]. Therefore, a critical care service system having quality care and safety for critically ill patients is essential for general surgery ICUs. Importance should be given to healthcare teams, particularly nursing personnel who look after patients closely for 24 h a day. Furthermore, health service system factors can lead to pre-cardiopulmonary arrest signs (pre-arrest), which refer to a critical condition in the body leading to circulatory and respiratory system disruption [12]. In this study the health system is based on the SEIPS model, where the objective is to design a working system that benefits both patient healthcare workers and the organization. The SEIPS includes patient outcomes such as patient safety and other dimensions of healthcare quality, as well as outcomes associated with healthcare workers and the organization. The health service system (patient, organization, personnel management, environment and technology, and instruments) defines (1) Person: The person could be the patient at the center of the system, a care provider, or another employee of a healthcare institution such as a biomedical engineer, a unit clerk. (i.e., physical characteristics, psychological characteristics, education, skill, knowledge, motivation, and need). (2) Organization: Different structurally related characteristics exist within the organization. Some examples include the hospital’s level, the number of their care models such as teamwork (physician, physician assistant, nurse practitioner, nurse’s aid, technical nurse, intensivists, clerk), and work schedule. (3) Task or Personnel management: There are characteristics of the task or jobs healthcare providers must execute, including what the tasks themselves are, as well as characteristics such as nurse characteristics, nursing workload, workflow, time pressure, and job control. Workload, according to conceptualization, is determined by the acuity and dependency of the patient. Workload measures are appropriate to use when comparing the workload levels of nurses with different specialties or job titles. (4) Environment: There are features of the environment in which healthcare providers work. The features include lighting, noise, and physical space and layout. (5) Technology and Instruments: The term uses various quantities and qualities of health information technologies, medical devices, and other tools and technologies; for example, the number and types of their technologies and their availability, unviability of the necessary equipment in a timely manner (e.g., electronic or paper health records, medical devices and monitoring, and equipment supplies [13,14].)

Elderly and critically ill patients require increased care [15,16,17,18]. By analyzing the factors leading to circulatory and respiratory downtime, the research framework to be used was developed based on the Smith and Carayon model, the System Engineering Initiative for Patient safety (SEIPS). According to the literature review, the factors in the health service system that are related to the quality care and safety of critically ill patients, in addition to affecting the quality of care for critically ill patients, include finding the leading cause of pre-cardiopulmonary arrest signs after surgery in critically ill patients, including personal or patient factors, organizational factors, task management factors, environmental factors and technology and tool factors [19,20,21,22,23].

In Thailand, a study conducted by Khunasathian et al. [24] revealed the factors influencing pre-cardiopulmonary arrest signs within the first 24 h after open heart surgery among 184 patients with an average score of pre-cardiopulmonary arrest signs 7.2 (SD + 1.6). A total of 183 cases (53.10%) had pre-cardiopulmonary arrest sign scores between 5 and 7, and 83 cases (41.70%) had pre-cardiopulmonary arrest sign scores exceeding 8 points, which is the highest risk score for pre-cardiopulmonary arrest signs. In addition, other studies reported that about 60% of patients who underwent cardiac surgery and had circulatory and cardiac arrest within the first 2 h after surgery showed abnormal circulatory system conditions [25]. 

The purpose of this study was to assess the incidence rate of an event leading to pre-cardiopulmonary arrest signs within the first 24 h after surgery among critical post-general surgery patients in ICUs, and to analyze the factors influencing the health service system (patient, organisation, personnel management, environment and technology, and instruments) and the patient care process in critical post-general surgery patients in the ICU.

## 2. Materials and Methods

### 2.1. Setting

Thailand is a country in Southeast Asia composed of 76 provinces and comprising several distinct geographies in the four regions: northern, north-eastern, central, and southern. The researcher randomly selected provinces with hospitals in the centre of each region. After randomly selecting medical centre hospitals from each province, 6 hospitals were selected for the study. Seven general hospitals were randomly selected. During the randomized selection of advanced tertiary hospitals from every region in Thailand, 2 hospitals were selected based on the ratio of medical centre hospitals to general hospitals in 2–3 provinces. The researcher obtained data from the following 6 provinces and medical centre hospitals. To calculate the sample size in this study, the researcher used 2-step sample size calculation and selected methods of higher sample calculation as follows: after calculation of the sample by determining the size and number of hospitals, the researcher randomly selected hospitals based on stratified random sampling by using hospital level (stratum) and all critically ill patients in the general surgery ICU (sampling unit supply). After calculating and sampling each hospital, the configuration was proportional to the size of the hospital based on the sampling according to probability proportional to size (PPS) [26].

For choosing a study area (regional level), the researcher was interested in studying the differences in patient factors and the health service system factors that provide different care for patients. The study area comprised health service facilities for critically ill patients treated in general surgery ICUs, hospital centers, general hospitals, and tertiary hospitals in Thailand. This study explored the critical care service system for critical patients treated in general surgery ICUs in Thailand. The inclusion criteria for the patient group were: (1) age ≥ 18 years; (2) patients admitted to general surgery in advance-level, standard-level, and super tertiary-level hospitals within a given period (all ICU admissions to a surgical ICU at a retrospective cohort time); and for nursing personnel group were: (1) professional nurses working in general surgery ICUs at the time of study; (2) full-time employment in the general surgery ward for more than one year. The sample consisted of 15 hospitals from all regions of Thailand, comprising the following: 2 super tertiary hospitals (n = 128, 33.0%); 6 advance hospitals (n = 160, 41.1%); and 7 standard hospitals (n = 101, 25.9%). The data were collected from 2018 to 2019. A total of 389 participants were selected from the record forms of post-general surgery patients in ICUs, and 233 nurses working in the general surgery ICUs who met the study’s inclusion criteria and completed the questionnaires for analysis were selected.

### 2.2. Measures

For critically ill patient assessments, we used the patients’ medical records. The research instrument used for data collection was an evaluation form created by the researcher based on a review of relevant literature on each factor with the following 6 sets of questionnaires—general information questionnaire on patient health information and surgery information (23 items). The tool passed the examination by 3 experts for the suitability of recording general information and clinical features with a content validity index (CVI) of 0.92.

The pre-cardiopulmonary sign scores assessment: Appropriateness of using data and clinical characteristics of patients [26] was judged by using the activation criteria for medical emergency teams [3]. Hodgetts and colleagues developed activation criteria for medical emergency teams to detect signs of pre-cardiopulmonary arrest in the early stages. The scores were evaluated for each of the following three sections: symptoms, physiology, and laboratory test results. The scores in all three sections were combined by scoring each level with the highest level, regardless of whether the patient had one or more symptoms together. The total score of the entire instrument had a minimum of 0 points (no pre-cardiopulmonary arrest) and a maximum of 11 points (severe pre-cardiopulmonary arrest). Content validity (CVI) was 0.85 in this case.

Information at the organizational level: It involves corporate research and is created by reviewing relevant literature, includes information about the name and province of the hospital, hospital level, total number of beds in the hospital, number of beds in the general surgery ward, number of critically ill surgical patients admitted to the general surgical wards per year, number of critically ill patients admitted to the general surgery ICU per month, number of critical care physicians in the general surgery ICU practices, and nursing care for critical patients (clinical practice guidelines (CPG) and patterns of multidisciplinary teams (23 questions]).

The instrument passed validation checking for the use of enterprise data records by 3 experts, in which the content validity index (CVI) was 0.91.

Information management personnel: It involves the assessment of personnel such as nursing staff for conducting research and is created using reviews of the relevant literature, includes information about the qualifications of the nursing staff who are different in terms of knowledge, specific expertise, and work experience (20 items). The instrument passed the examination of suitability for recording the characteristics of nursing personnel by 3 experts, in which the content validity index (CVI) was 1.

Questionnaire about work obstacles in the work of nurses in the intensive care unit (ICU Nurse Questionnaire), a questionnaire previously developed by Gurses and Carayon [27] was used in this study. Questions were asked about the work view and the environmental and working conditions of nursing personnel working on duty. There were 44 questions with four-parts measurement. In regard to instrument reliability, an international study among professional nurses found reliability of scores (Cronbach’s alpha for internal consistency) in every area to be 0.91 [28]. In Thailand, no study has been conducted on navigation instruments concerning obstacles in the work of nurses in the ICU (Performance Obstacles of ICU Nurse Questionnaire). Therefore, the researcher acquired permission from the owner of the above-mentioned instrument to use it and then translated it into Thai through the processes of forward (English to Thai) and backward (Thai to English) translation. The instrument was tested for content validity, its CVI was 0.97, and its reliability was determined by the approval from the Ethics Committee on Research Involving Human Subjects.

Questionnaire for knowledge and skills in nursing practice of nurses working in critical care: Instruments used in this study were divided into two topics nursing knowledge and skills of critical unit professionals. In this study, the researcher used an effective questionnaire on nursing knowledge and skills created by Manatnan Nakkerd from a study on “Morale in Critical Unit Professional Nursing Work and Measurement of Nursing Efficiency in Critical Units” [29]. The questionnaire contained 56 questions on the following seven aspects. Responses were rated on a 5-level rating scale. The scores resulting from technology questionnaires in the general surgery ward were interpreted from the highest to the lowest level using the following average scores. The instrument was tested for a content validity index (CVI) of 0.97.

Instruments and Technology in General Surgery ICUs: The instruments for research in technology and instruments in the general surgery ward for this study included a questionnaire based on textbooks, journals, and related literature searched by the researcher. The characteristics of technology and the number of instruments used in the service of critically ill patients in each organisation, such as the number, type, modernity and complexity of the instruments used for their observation, surveillance, monitoring, and treatment (different device) (MDE) to be able assess the threat to the lives of patients (Items 1 to 4 above) were studied. The scores resulting from technology questionnaires in the general surgery ward were interpreted from the highest to the lowest level using the following average scores. The instrument passed the examination of suitability for recording nursing personnel characteristics by 3 qualified experts, with a content validity index (CVI) of 1.

The authors applied the translation processes of the forward and back-translation methods for the WHO guidelines for personnel, task level assessment, and work obstacles in the work of nurses in the intensive care unit (ICU Nurse Questionnaire).

### 2.3. Statistical Analysis

Descriptive statistics were used to analyze basic data and clinical features of samples in each study unit. For data analysis, a single value (univariable analysis) was used accordingly. Continuous variables are presented in the form of percentages as well as in mean and standard deviation. General analysis of the sample data evaluated relationships with chi-square testing and Pearson’s product-moment correlation coefficient at each level of factor, such as patient factors, nursing personnel factors, organizational factors, technological and tools factors, and environmental factors. The researcher then determined the relationships among the factors in the health service system and the patient care process based on the conditions that lead to disruption of the circulatory and respiratory systems in critically ill patients in general surgery ICUs. The researcher also identified interaction effects accordingly.

The odds ratio was calculated, and chi-square statistics were used to analyze the statistical significance between the independent and dependent variables in the final model to determine consistency with regression analysis. Multilevel mixed method repeated measure was used to predict the dependent variable using coefficients, log-likelihood (LL), Akaike information criterion (AIC), and consistency of the independent variables. In this study, pre-cardiopulmonary arrest sign scores binary outcomes defined high pre-cardiopulmonary arrest sign with scores over 4 points, and low pre-cardiopulmonary arrest sign with scores 1–4 points [3,24].

Analysis of the correlation coefficient was performed between the independent variables, such as patient factors, nursing personnel factors, organizational factors, information technology, and environmental factors, which were different in each organization. The dependent variables were the rate of pre-cardiopulmonary arrest signs, and occurred in the form of disruption of the circulatory and respiratory systems within the first 24 h after surgery by analyzing the level to find the relationships, using descriptive statistics and statistical analysis for structural relationship analysis (multilevel mixed method: repeated measure) by STATA version 13.030-student Stata lab perpetual license: Serial number: 401306230357 licensed to USER14 Faculty of Medicine Vajira Hospital.

## 3. Results

### 3.1. Incidence Rates of Pre-Cardiopulmonary Arrest Signs

The incidence rate of pre-cardiopulmonary arrest signs indicated by scores in critical post-general surgery patients was 49.05 per 1000 person-hours. The most common pre-cardiopulmonary arrest signs were hemodynamic instability, respiratory instability, and neurological alterations. High scores for pre-cardiopulmonary arrest signs were significantly associated with the time for high pre-cardiopulmonary arrest sign event measurement at 0–24 h, which decreased every hour (β = 4.820, *p* < 0.001) when compared with low pre-cardiopulmonary arrest sign score significance.

### 3.2. The Probability of High-Risk Pre-Cardiopulmonary Arrest Signs

**Figure 1:**
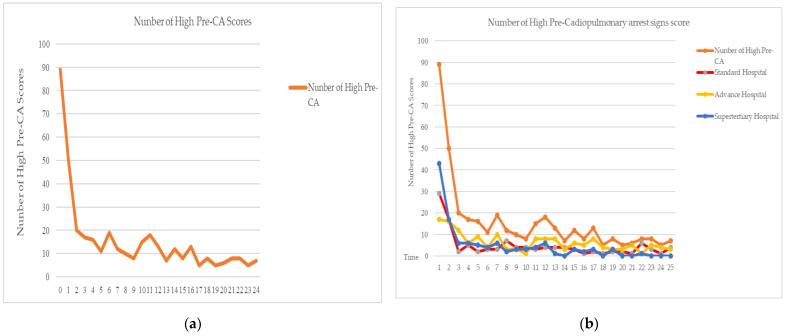
(**a**) Mean pre-cardiopulmonary arrest signs over time within 0–24 h. (**b**) The mean pre-cardiopulmonary arrest sign scores across time for three hospital levels.

The result on the probability of patients having high-risk pre-cardiopulmonary arrest signs over time. This is a measure of time within 0–24 h following surgery. The results showed pre-cardiopulmonary arrest sign scores in critical post-general surgery patients’ dropdowns at time 0–5 h. After adjusting for the estimated pre-cardiopulmonary arrest sign scores in post-general surgery patients, high pre-cardiopulmonary scores fluctuated over time and increased from time 5–6 h. There were variations in the pre-cardiopulmonary arrest sign scores in post-general surgery patients, which fluctuated again at time from 9–24 h, as shown in Figure 1a.

The probability of patients having high risk pre-cardiopulmonary arrest signs changed over time between hospital levels as presented in Figure 1b—the mean pre-cardiopulmonary arrest sign scores over time for the 3 hospital levels. Generally, the mean pre-cardiopulmonary arrest sign scores in critical post-general surgery patients were highest at time 1 h, with responses dropping over time at all hospitals. According to the findings, time 1–3 h had high scores, which were even more prevalent at the super-tertiary hospital level. Furthermore, there were decreases in pre-cardiopulmonary arrest sign scores over time. The advanced hospital level had more pre-cardiopulmonary arrest sign score fluctuations than the super-tertiary and standard hospital levels. The differences in pre-cardiopulmonary arrest sign scores at all hospital levels are of interest for exploring the factors influencing pre-cardiopulmonary arrest signs.

More men than women (57.6% and 42.4%, respectively) showed pre-cardiopulmonary arrest signs. The average age was approximately 59.8 (SD ± 17.5) years (range, 18–99 years). Furthermore, the most critical post-general surgery patients were older than 71 years (29.8%) (Table 1).

The multi-level mixed model data with pre-cardiopulmonary arrest signs between the patient and hospital level. Nevertheless, the highest scores for the pre-cardiopulmonary arrest signs were noted for patients aged 18–40 years, indicating that patient age had negative direct effects on pre-cardiopulmonary arrest signs (OR = 0.708, 95% CI 0.525–0.956) (Table 2).

### 3.3. Factors Related the Pre-Cardiopulmonary Arrest Signs

Concerning surgical status (emergency, urgent, or elective), urgent surgeries were associated with an increased risk of infection and sepsis in surgical patients. Slightly over half (50.6%) of the patients’ operation statuses were emergency cases at all hospital levels, followed by elective and urgent cases in that order. Therefore, emergency operation status had significant negative direct effects on pre-cardiopulmonary arrest signs (OR = 0.519, 95% CI 0.356–0.756), followed by elective operation status (OR = 0.466, 95% CI 0.257–0.845).

Thus, the findings revealed that two of the patients’ dimensional factors (age and operation status) had significant positive direct effects on high-risk pre-cardiopulmonary arrest signs in. In addition, elective operation status and patient age of more than 61 years (OR = 2.936, 95% CI 1.113–7.747) and patient age of 18–40 years (OR = 2.163, 95% CI 1.060–4.414) were more significant factors, while the other two factors of patient age and operation status had no significant effects.

Hospital factors [hospital level] directly affected the pre-cardiopulmonary arrest signs of critical post-general surgery patients in the ICU within the first 24 h after surgery. The results indicated that one variable involving nurse allocation (RN:PN:NA) (OR = 27.889, 95% CI 3.497–222.380) had positive direct effects on the pre-cardiopulmonary arrest signs of critical post-general surgery patients in the ICU within the first 24 h after surgery.

Task factors, nurse-to-patients ratio directly affected the pre-cardiopulmonary arrest signs of critical post-general surgery patients in the ICU within the first 24 h after surgery. According to the findings, nurse-to-patient ratios of 1:1 and 1:2 (OR = 0.705, 95% CI 0.564–0.880, OR = 19.695, 95% CI 2.351–164.937) had positive direct effects on the pre-cardiopulmonary arrest signs of critical post-general surgery patients in the ICU within the first 24 h after surgery.

The factors in the patient care process had no significant direct effects on the pre-cardiopulmonary arrest signs of critical post-general surgery patients in the ICUs within the first 24 h after surgery. The findings of this study revealed the direct effects of the care process on high-risk pre-cardiopulmonary arrest sign scores. However, the results also presented different assessment instruments in the guidelines for the care of sepsis and shock in each hospital (SOS, SOFA, and APACHE) [30,31,32,33]. The findings also revealed that effective treatment of sepsis should involve rapid response teams (RRT). The results showed close adherence to the guidelines and the availability of rapid response teams at 46.67%. After interviewing nursing care providers involved in the care process of sepsis guidelines, it was revealed that staffing included multidisciplinary teams at only two super-tertiary hospitals and, surprisingly, at one standard hospital. Furthermore, the medical teams included rapid response teams that followed sepsis guidelines at three advanced hospitals and one standard hospital.

### 3.4. The Multi-Level Mixed Model of Pre-Cardiopulmonary Arrest Signs

The multi-level mixed model with repeated measure data logistic regression provided cluster-specific measures of associations (ORs) for the patient- and hospital-level variables reported in Table 2 [34]. Therefore, the results could be interpreted as an odds ratio within-cluster comparison. This study revealed hospital-adjusted associations between patient-level variables and pre-cardiopulmonary arrest signs within 24 h after general surgery. In Model 2, patient-level factors showed an increase in the positive aspect of pre-cardiopulmonary arrest signs when there were changes over time. Model 3 included interactions between operation status and age-adjusted level, with significance in elective case interactions with patients aged 18–40 years and elective case interactions with patients aged > 61 years. (OR = 2.163, 95% CI 1.060–4.414 and OR = 2.936, 95% CI 1.113–7.747, respectively.)

### 3.5. Comparison Model

The patient factors associated with high pre-cardiopulmonary arrest sign scores were as follows: (1) age of 18–40 years among critical post-general surgery patients (β = −0.343, *p*-value 0.025); (2) operation status: emergency surgery (β = −0.655, *p*-value 0.001), elective surgery (β = −0.763, *p* = 0.012) in relation to urgent surgery; and (3) the interaction of operation status and age of critical post-general surgery patients; elective surgery * age 18–40 years (β = 0.771, *p*-value 0.034) and elective surgery * age more than 61 years (β = 1.077, *p*-value 0.030). The organization factors associated with nurse allocation (staff mix) RN:PN:NA were (β = 3.328, *p*-value 0.002) compared with RN:NA. The personal factors associated with the nurse-to-patient ratio were as follows: (1) nurse-to-patient ratio of 1:2 (β = −0.349, *p* = 0.002); and (2) nurse-to-patient ratio of 1:3 (β = 2.980, *p* = 0.006) was more statistically significant than of a nurse-to-patient ratio of 1:1.

## 4. Discussion

In the current study, the incidence rate of pre-cardiopulmonary arrest signs in postoperative patients, was 49.05 per 1000 person-hours. The incidence of pre-cardiopulmonary arrest signs fluctuates over time. The mean time of a high proportion of pre-cardiopulmonary arrest sign score peaks at 0–2 h postoperatively. This study was performed in a critical care service system, which may imply that early recognition and intervention of cardiopulmonary arrest is essential to increase patient safety and QOC in the critical care service system. A recent study in Thailand, a retrospective cohort study from a large population, identified the incidence of in-hospital postoperative cardiac arrest as 12 patients per 10,000 surgeries (95% CI 6.20–21.00; *p* < 0.0001) [35].

In this study, the findings were similar for individual patients who had high pre-cardiopulmonary arrest sign scores, which fluctuated simultaneously in continuous outcomes of high pre-cardiopulmonary arrest sign scores within the first 24 h post-general surgery in the ICU. Nevertheless, the present study examined the outcomes by classifying high risk for high pre-cardiopulmonary arrest sign scores compared with low risk for pre-cardiopulmonary arrest sign scores as a binary outcome. The findings obtained can be used to promote better care for patients undergoing critical post-general surgery in terms of giving importance to the type of intensive care required by this group of patients within the first 24 h. Furthermore, this finding may be explained by the availability of better monitoring and skilled specialty care in ICUs. In Thailand, one study on the incidence rate of pre-cardiopulmonary arrest signs scores by Suchayada Kunstian collected data from 194 patients undergoing open heart surgery at a university hospital in Bangkok, Thailand. According to the findings of the present study on pre-cardiopulmonary arrest sign scores within the first 24 h after open-heart surgery. Most of the sample was male (55.7%) with an average age of 59.9 years, and 41.7% had severe pre-cardiopulmonary arrest signs scores (≥8 points). The average highest score of the sample was 7.2 (SD + 1.6) points. The findings confirmed those undergoing open-heart surgery always need intensive monitoring due to the risk for cardiopulmonary arrest, especially within 24 h post-surgery [24].

Most of the factors were old age, high ASA classification, poor function status, renal failure, cardiothoracic surgery, emergency operation, and preoperative sepsis [2,35,36,37]. In this study, the factors influencing the occurrence of high-risk pre-cardiopulmonary arrest sign scores were found to be patients’ dimensions [age; 18–40 years, operation status; emergency surgery, and elective surgery]. (OR = 0.708, 0.519, 0.466, respectively) *p*-value < 0.05. 

Therefore, the finding from the modified model was goodness-of-fit with the existing data greater than that of hypothesized model. The results rejected the theoretical hypothesis and accepted the new model. The findings revealed that the top three factors in the health service system, significantly influencing the occurrence of pre-cardiopulmonary arrest signs, were nurse allocation (nurse-to-patient ratio of 1:3) and elective surgery interaction with ages more than 61 years (OR = 27.889, 19.695, 2.936, respectively) (*p* < 0.05), as shown in Table 2.

The discussion of the SEIPS model for a modified model of quality of care in critical post-general surgery patients in ICUs composed of model constructs, model fit, and relationships between the constructs of the theoretical model is presented as follows: The findings show the modified model of quality of care in critical post-general surgery patients with better fit indices of Intra-class correlations follows: ICC = 0.033, Akaike’s information criterion: AIC = 3495.218, Log-likelihood: LL = −1733.608, and Chi-square = 0.038 (Table 3). This multilevel study contributes to the development of nursing science by providing evidence that nurses are significant healthcare providers in promoting positive health outcomes in critical post-general surgery patients. The results show how healthcare services and patient factors affect the health of Thai patients in critical condition. Based on the findings, suggestions have been made for further advancement of the discipline of nursing as follows: training course in critical nurse, effective monitoring system, as well as clinician skills [38].

### Strength and Limitations

The findings of this study will enhance our understanding of the best model for critical care service systems. The final model from this study aimed to identify the most important factors affecting the pre-cardiopulmonary arrest sign in post-general surgery patients. A focused collection of key elements in the entire process of surgical service from surgical resources through surgical performance among post-general surgery patients. 

The findings from this study suggest that pre-cardiopulmonary arrest signs events occur in extreme among post-general surgery elderly patients undergoing surgery elective surgery. Since the work system and content of the task of nurses have a wide range and extreme predetermined framework, surgical nurses need to anticipate and recognise unexpected problem. Preparedness for the unexpected requires critical knowledge, as well as the hardness of non-human and human resources. Nurses should be prepared to manage this high-risk group of surgical patients promptly.

## 5. Conclusions

First, this study has provided new knowledge regarding the incidence rate of pre-cardiopulmonary arrest and multilevel factors influencing pre-cardiopulmonary arrest signs among critical post-general surgery patients in ICUs in Thailand. Second, the findings can be used as baseline data for the improvement and design of health service deliveries, allocation of healthcare providers, and implementation of standard guidelines for the care of critical post-general surgery patients in ICUs. Third, the results indicate that the healthcare system in Thailand is mainly concerned with physical function, but it is not sensitive to rapid response to deterioration signs in critical patients, thereby leading to poor quality of care (QOC) for most of the patients in this study. 

## 6. Recommendations

Based on the study findings, the following recommendations are proposed regarding nursing science health policies for organizational management, nursing practice, service delivery, and further research. To improve the quality of care for critical post-general surgery patients, critical care service delivery should be delegated to nurses through nurse allocation and critical care nursing training. Furthermore, patients should receive rapid responses to pre-cardiopulmonary arrest signs. Guidelines must be established for critically ill post-general surgery patient care, particularly for elderly patients who underwent elective surgery.

## Figures and Tables

**Table 1:** Characteristics of critical post-general surgery patients during first 24 h.

Characteristics (n = 389)		Total	
		Number	Percent
Gender			
	Male	224	57.6
	Female	165	42.4
Age			
	18–40 years	57	14.6
	41–60 years	132	34.0
	61 years and above	200	51.4
	Mean Age in Years ± SD (age range)	59.8 ± 17.5	(18–99)
Marital Status			
	(a) Married	205	52.7
	(b) Single	146	37.5
	(c) Divorced	38	9.8
Admission source			
	(a) Emergency Department	95	24.4
	(b) Ward	260	66.9
	(c) Referral from other hospital	34	8.7
	(d) Operation room	0	0
Operation Status			
	(a) Urgent	60	15.5
	(b) Emergency	197	50.6
	(c) Elective	132	33.9
Discharge Source			
	(a) Ward	321	82.5
	(b) ICU	68	17.5
Discharge Status			
	(a) Complete Recovery	10	2.6
	(b) Improve	297	76.3
	(c) No Improvement	22	5.7
	(d) Death	60	15.4
CCI Score (Charlson Comorbidity Index)			
	(a) Mild (1–2 points)	122	31.4
	(b) Moderate (3–4 points)	119	30.5
	(c) Severe (more than 5 points)	80	20.6
	(d) None	68	17.5
ASA Score (American Society of Anesthesiologists)			
	(a) ASA I	11	2.8
	(b) ASA II	61	15.7
	(c) ASA III	245	63.0
	(d) ASA IV	71	18.3
	(e) ASA V	1	0.3
SAS Score (Surgical Apgar Score)			
	(a) Minimal risk (9–10 points)	4	1.1
	(b) Low risk (7–8 points)	30	7.7
	(c) Medium risk (5–6 points)	141	36.2
	(d) High risk (0–4 points)	214	55.0
Primary Diagnosis			
	(a) CA	107	27.5
	(b) Duodenal Ulcers	54	13.9
	(c) Neurology	31	8.0
	(d) Abdominal Injury	30	7.7
	(e) Urology	24	6.2
	(f) Wound	21	5.4
	(g) Cholecystitis	18	4.6
	(h) CBD Stone	18	4.6
	(i) Pelvic Fracture	15	3.9
	(j) Other (cirrhosis, pericardial.effusion, morbid obesity)	71	18.2
Operation Procedure			
	(a) Laparotomy	211	54.3
	(b) Orthopedic	50	12.9
	(c) Neurology	35	9.0
	(d) Gastrectomy	20	5.1
	(e) Gastroscopy	20	5.1
	(f) Pancreatomy	18	4.6
	(g) Debridement	18	4.6
	(h) Vascular	5	1.3
	(i) Other (tracheostomy, hemorrhoidectomy, herniectomy)	12	3.1

**Table 2:** Estimated odds ratio and variance components for the multi-level mixed model and the repeated measure data logistic regression model.

Variable	Model 2 Odd Ratio *(*95% CI*)*	Model 3 Odd Ratio *(*95% CI*)*
Time 0–24 variance	0.889 *** (0.876 to 0.903)	0.889 *** (0.875 to 0.903)
Operation Status		
Emergency	0.648 *** (0.509 to 0.824)	0.519 * (0.356 to 0.756)
Elective	0.832 (0.604 to 1.147)	0.466 * (0.257 to 0.845)
Age Adjusted Level		
18–40 years	0.860 (0.685 to 1.069)	0.708 * (0.525 to 0.956)
More than 61 years	0.995 (0.728 to 1.359)	0.767 (0.529 to 1.113)
Nurse allocation		
RN:PN:NA	26.142 * (3.268 to 209.088)	27.889 * (3.497 to 222.380)
Nurse-to-Patient ratio		
1:2	0.707 * (0.564 to 0.886)	0.705 * (0.564 to 0.880)
1:3	19.074 * (2.265 to 160.581)	19.695 * (2.351 to 164.957)
Operation Status # Age Adjusted Level		
Emergency # 18–40 years		1.336 (0.810 to 2.203)
Emergency # More than 61 years		1.865 (0.855 to 4.060)
Elective # 18–40 years		2.163 * (1.060 to 4.414)
Elective # More than 61 years		2.936 * (1.113 to 7.747)

* significant at *p* < 0.05, *** significant at *p* < 0.001. OR = odds ratio, CI = confidence interval.

**Table 3:** Comparison of Models.

Model	ICC	AIC	−Log Likelihood	Chi-Square
Main model (M_0_) (n = 9725)	0.058	3800.924	−1765.343	0.0010
M_1_ model: Add repeated time 0–24 (n = 9725) Logit $\hat{y\;}$ = −4.602 Time for pre-cardiopulmonary arrest signs_1_	0.070	3536.687	−1765.343	0.0002
M2 model: Fixed effect model; add predicted variable (n = 9725) Logit $\hat{y\;}$ = −4.602 Time of pre-cardiopulmonary arrest signs _1_*** − 0.343 patients aged 18–40 years _2_* − 0.264 patients aged more than 61 years _3_ − 0.655 emergency operation status _4_*** − 0.762 elective operation status _5_* + 3.328 nurses’ allocation (RN:PN:NA) _6_* − 0.-349 nurse-to-patients ratio (1:2) _7_* + 2.980 nurse-to-patients ratio (1:3) _8_*	0.040	3494.924	−1737.462	0.0176
M3 model: intercept effect model; add interaction effect variable (n = 9725) Logit $\hat{y\;}$ = −4.602 Time of pre-cardiopulmonary arrest signs _1_*** − 0.343 patients aged 18–40 years _2_* − 0.264 patients aged more than 61 years _3_ − 0.655 emergency operation status _4_*** − 0.762 elective operation status _5_* + 3.328 nurses allocation(RN:PN:NA) _6_* − 0.−349 nurse-to-patients ratio (1:2) _7_* + 2.980 nurse-to-patients ratio (1:3) _8_* + 0.289 emergency operation status# patient aged 18–40 years _9_ + 0.623 emergency operation status# patient aged over 61 years _10_ + 0.771 elective operation status# patients aged 18–40 years _11_* + 0.967 elective operation status# patient age more than 61 years _12_	0.033	3495.218	−1733.608	0.0384

* significant at *p* < 0.05, *** significant at *p* < 0.001.

## Data Availability

Not applicable.
